# Enzalutamide promotes an early plasticity-associated transcriptional state without terminal neuroendocrine differentiation in prostate cancer cells

**DOI:** 10.3389/fonc.2026.1870253

**Published:** 2026-07-24

**Authors:** Ryuta Watanabe, Mami Chosei, Haruna Arai, Noriyoshi Miura, Tadahiko Kikugawa, Takashi Saika

**Affiliations:** 1 Department of Urology, Ehime University Graduate School of Medicine, Toon, Japan; 2 Department of Biochemistry and Molecular Genetics, Ehime University Graduate School of Medicine, Toon, Japan

**Keywords:** androgen receptor pathway inhibition, lineage plasticity, neuroendocrine prostate cancer, transcriptional adaptation, transcriptomic reprogramming

## Abstract

**Introduction:**

Neuroendocrine prostate cancer (NEPC) is an aggressive treatment-associated lineage state emerging with potent androgen receptor (AR) pathway inhibition. Although treatment-emergent NEPC is increasingly recognized, the transcriptional consequences of sustained AR suppression remain incompletely defined. It remains unclear to what extent AR-targeted therapies reshape cellular identity and engage neuroendocrine-associated transcriptional programs without full lineage-defining differentiation.

**Materials and methods:**

Bulk RNA sequencing was performed in AR-dependent LNCaP and castration-resistant C4–2 prostate cancer cells under untreated conditions, androgen deprivation, first-generation AR antagonism with bicalutamide, and second-generation AR pathway inhibition with enzalutamide, using NCI-H660 as a NEPC reference. Transcriptomic organization and pathway-level changes were assessed using principal component analysis, comparative analyses, module score analysis of lineage-associated programs, and Gene Ontology–based functional enrichment.

**Results:**

In LNCaP cells, AR suppression induced a progressive transcriptomic shift toward the NEPC reference cell line H660, with this trend being most pronounced in enzalutamide-treated cells. Canonical AR target genes (KLK3, TMPRSS2, NKX3-1, and FKBP5) were suppressed, whereas neuroendocrine-associated genes (NCAM1, ENO2, PEG10, and DLL3) showed partial induction. Canonical NEPC markers and lineage-defining transcription factors, including CHGA, INSM1, and SOX2, were not activated. Module score analysis demonstrated selective activation of lineage plasticity–associated programs corresponding to an intermediate Phase 2 state without engagement of canonical neuroendocrine differentiation programs. Gene-wise analyses further showed preferential induction of developmental and plasticity-associated transcriptional programs by enzalutamide compared with bicalutamide. In contrast, C4–2 cells exhibited context-dependent and incomplete neuroendocrine-associated transcriptional changes, consistent with modulation of a pre-existing permissive transcriptional state rather than stable neuroendocrine differentiation.

**Conclusions:**

Potent AR pathway inhibition does not induce terminal neuroendocrine differentiation *in vitro* but promotes an adaptive plastic intermediate state characterized by partial activation of neuroendocrine-associated transcriptional programs. These findings suggest that enzalutamide preferentially enhances early lineage plasticity rather than bona fide NEPC differentiation and provide a framework for distinguishing adaptive transcriptional reprogramming from true neuroendocrine lineage conversion in treatment-emergent prostate cancer.

## Introduction

Androgen receptor (AR) signaling plays a central role in prostate cancer progression, and androgen deprivation therapy (ADT) has long been the cornerstone of treatment for advanced disease. The introduction of second-generation AR pathway inhibitors, such as enzalutamide, has significantly improved survival in metastatic castration-resistant prostate cancer (CRPC) (1, 2); however, resistance to AR-targeted therapies almost inevitably emerges (3).

A clinically important resistance mechanism is neuroendocrine prostate cancer (NEPC), an aggressive and treatment-refractory subtype characterized by loss of AR dependency and acquisition of neuroendocrine features (4, 5). NEPC is associated with rapid progression, frequent visceral metastases, and poor prognosis, and is typically treated with platinum-based chemotherapy with limited durability (5, 6).

Accumulating evidence suggests that NEPC can arise in the context of treatment-induced lineage plasticity rather than expansion of pre-existing neuroendocrine cells (4, 7). In the era of potent AR pathway inhibition, treatment-emergent NEPC and related AR-independent phenotypes account for approximately 15–20% of advanced CRPC cases in Western cohorts (8–10).

These observations raise an important question: how does sustained AR suppression reshape cellular identity at the transcriptional level? Although AR signaling can be suppressed by androgen deprivation, first-generation AR antagonists, and second-generation AR pathway inhibitors, whether these approaches exert distinct effects on transcriptional organization and lineage-associated programs remains incompletely understood (4, 7). Furthermore, the intermediate transcriptional adaptations that occur before stable neuroendocrine lineage conversion remain poorly characterized. It is unclear whether prolonged AR suppression directly promotes canonical neuroendocrine differentiation or instead induces a plastic transitional state marked by partial activation of neuroendocrine-associated programs.

Here, we investigated the transcriptional consequences of long-term AR suppression in androgen-dependent prostate cancer cells using distinct therapeutic modalities. By comparing untreated, androgen-deprived, bicalutamide-treated, and enzalutamide-treated cells with a neuroendocrine prostate cancer reference line, we examined whether potent AR inhibition engages neuroendocrine-associated transcriptional programs in the absence of terminal neuroendocrine differentiation. We further sought to determine whether different modes of AR suppression produce distinct adaptive transcriptional states, thereby providing insight into early lineage plasticity preceding bona fide neuroendocrine transformation.

## Materials and methods

### Cell culture and generation of long-term AR-suppressed cell populations

#### Cell culture and generation of long-term AR-suppressed cell populations

**Figure 1:**
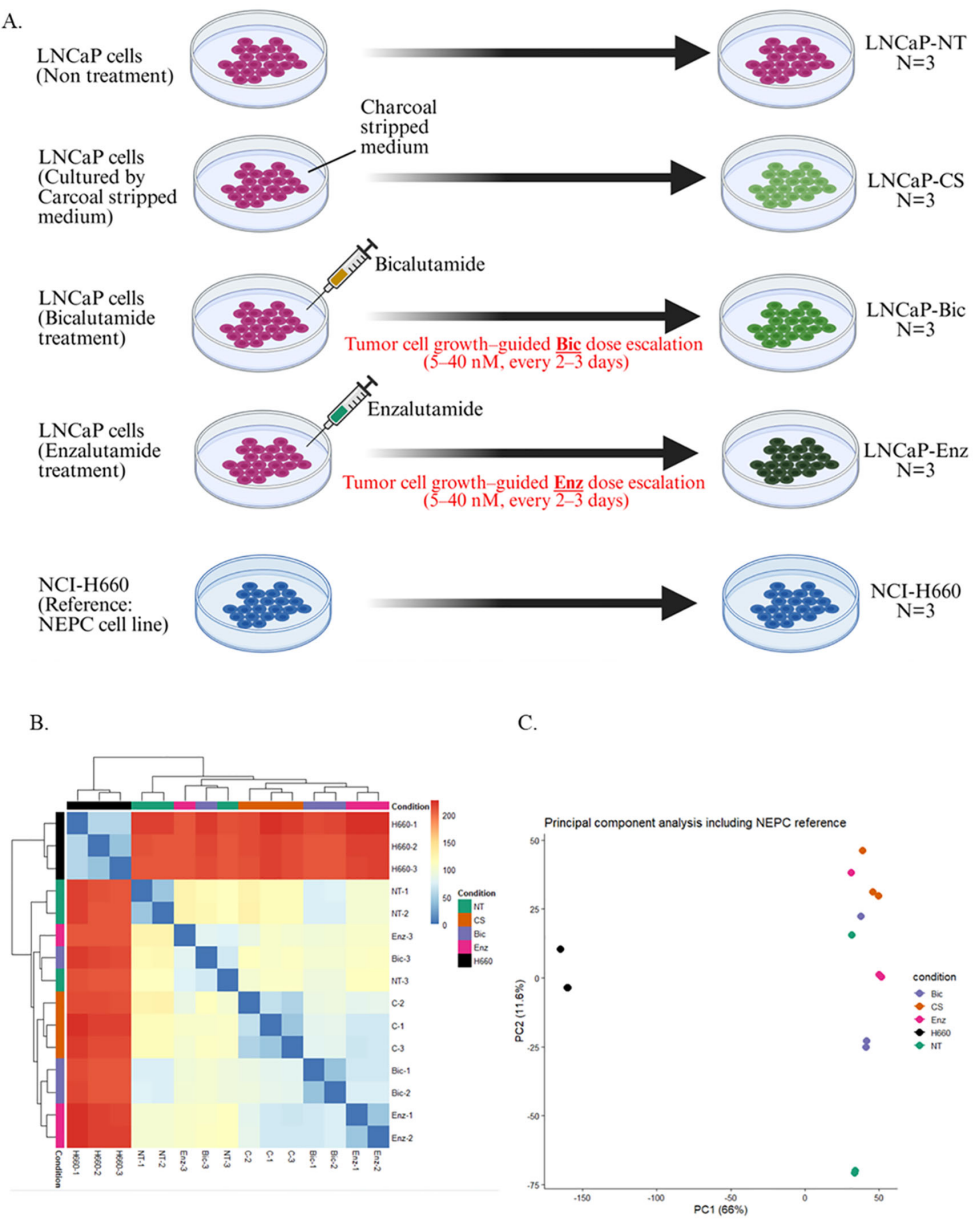
Experimental design for generation of long-term AR-suppressed LNCaP cell populations. **(A)** Experimental design for stepwise induction of AR inhibitor–resistant LNCaP cell lines. LNCaP cells were cultured in charcoal-stripped serum and exposed to gradually increasing concentrations of bicalutamide or enzalutamide, starting at 5 nM. Cell proliferation was monitored every 2–3 days, and drug concentrations were increased in a stepwise manner (5, 10, 20, and 40 nM) only after cells resumed growth following transient growth arrest. This stepwise dose-escalation procedure was repeated until cells reached a stable adapted state under continuous AR-suppressive conditions. Experiments were performed independently in triplicate (n = 3). H660 cells were included as a reference neuroendocrine prostate cancer (NEPC) model. This schematic was created using BioRender (https://www.biorender.com/). **(B)** Sample-to-sample distance heatmap of RNA-seq data. Euclidean distances were calculated using variance-stabilized gene expression values. Hierarchical clustering shows grouping of biological replicates within each condition and separation of the NEPC reference cell line H660 from LNCaP-derived samples. **(C)** Principal component analysis (PCA) of transcriptomic profiles under stepwise AR inhibition. PCA based on variance-stabilized gene expression values shows the distribution of untreated (NT), charcoal-stripped serum (CS), bicalutamide-treated (Bic), enzalutamide-treated (Enz), and H660 samples in the space defined by the first two principal components.

Human prostate cancer cell lines LNCaP (ATCC CRL-1740), C4-2 (ATCC CRL-3314), and NCI-H660 (ATCC CRL-5813) were obtained from the American Type Culture Collection (ATCC, Manassas, VA, USA). LNCaP and C4–2 cells were maintained at 37 °C with 5% CO_2_ in RPMI-1640 medium supplemented with 10% fetal bovine serum (FBS), 20 U/mL penicillin, and 100 µg/mL streptomycin (**Figure 1**).

To model early transcriptional adaptation to prolonged androgen receptor (AR) suppression, LNCaP and C4–2 cells were cultured under four conditions: untreated control (NT), androgen deprivation using charcoal-stripped serum (CS), long-term bicalutamide exposure (Bic), and long-term enzalutamide exposure (Enz). Bicalutamide (Selleck Chemicals, Houston, TX, USA; Cat. No. S1190) and enzalutamide (ChemScene, Monmouth Junction, NJ, USA; Cat. No. CS-0317) were dissolved in DMSO and used for all experiments. For the Bic and Enz conditions, cells were initially exposed to 5 nM of each drug, and drug concentrations were increased in a stepwise manner (5, 10, 20, and 40 nM) every 2–3 days according to cell growth status. Dose escalation was performed only after transient growth arrest followed by partial recovery or stabilization of cell growth was observed. At the final concentration, cells exhibited minimal net growth while remaining stably maintained in culture; RNA was collected at this plateau-like adapted state. As a result, the adaptation process typically required approximately 3–4 weeks, although the exact duration varied according to cell growth dynamics. Thus, these populations were regarded as AR-suppression–adapted cells rather than fully established drug-resistant derivative cell lines. Experiments were performed independently in biological triplicate.

The neuroendocrine prostate cancer cell line NCI-H660 was maintained as a suspension culture under specialized conditions. Cells were cultured at 37 °C with 5% CO_2_ in DMEM/F-12 (1:1) medium supplemented with 5% FBS, GlutaMAX (1×), and ITS-G supplement (1×). The medium was further supplemented with hydrocortisone and β-estradiol at final concentrations of 10 nM each. These supplements were prepared from stock solutions and added during medium preparation at a scale of 500 mL. NCI-H660 cells were typically cultured in approximately 10 mL of medium per culture vessel. Because the cells grow in suspension and are sensitive to mechanical stress, medium exchange was performed by gentle centrifugation followed by careful removal of the supernatant and resuspension in fresh medium. Complete medium replacement was typically performed every 1–2 weeks, depending on cell density and viability. Due to the fragile growth characteristics of NCI-H660 cells, special care was taken during handling and medium replacement.

### RNA extraction and RNA sequencing

Total RNA was extracted from cultured cells using standard phenol–chloroform–based or column-based methods according to the manufacturer’s instructions. RNA quality and integrity were assessed prior to library preparation. For the Bic and Enz conditions, RNA was collected 72 h after cells had reached the final drug concentration (40 nM) and stabilized under continuous drug exposure. For the NT and CS conditions, RNA was collected at the corresponding experimental endpoint.

RNA sequencing libraries were prepared using poly(A)-selected RNA and sequenced on an Illumina platform to generate paired-end reads. Sequencing was performed for three biological replicates per condition. LNCaP analyses included NT, CS, Bic, Enz, and H660 samples (n = 3 each), whereas C4–2 analyses included NT, CS, Bic, and Enz samples (n = 3 each).

### RNA-seq data processing and normalization

Raw sequencing reads were quantified at the transcript level using Salmon, with alignment-free quasi-mapping to the human reference transcriptome. Transcript-level abundance estimates were imported into R using the tximport package to obtain gene-level count matrices.

Differential expression analysis and normalization were performed using DESeq2. Variance-stabilizing transformation (VST) was applied to normalized count data for downstream analyses. VST-normalized expression values were used for principal component analysis (PCA), sample-to-sample distance calculations, and all downstream visualization analyses.

### Signature gene sets

**Figure 2:**
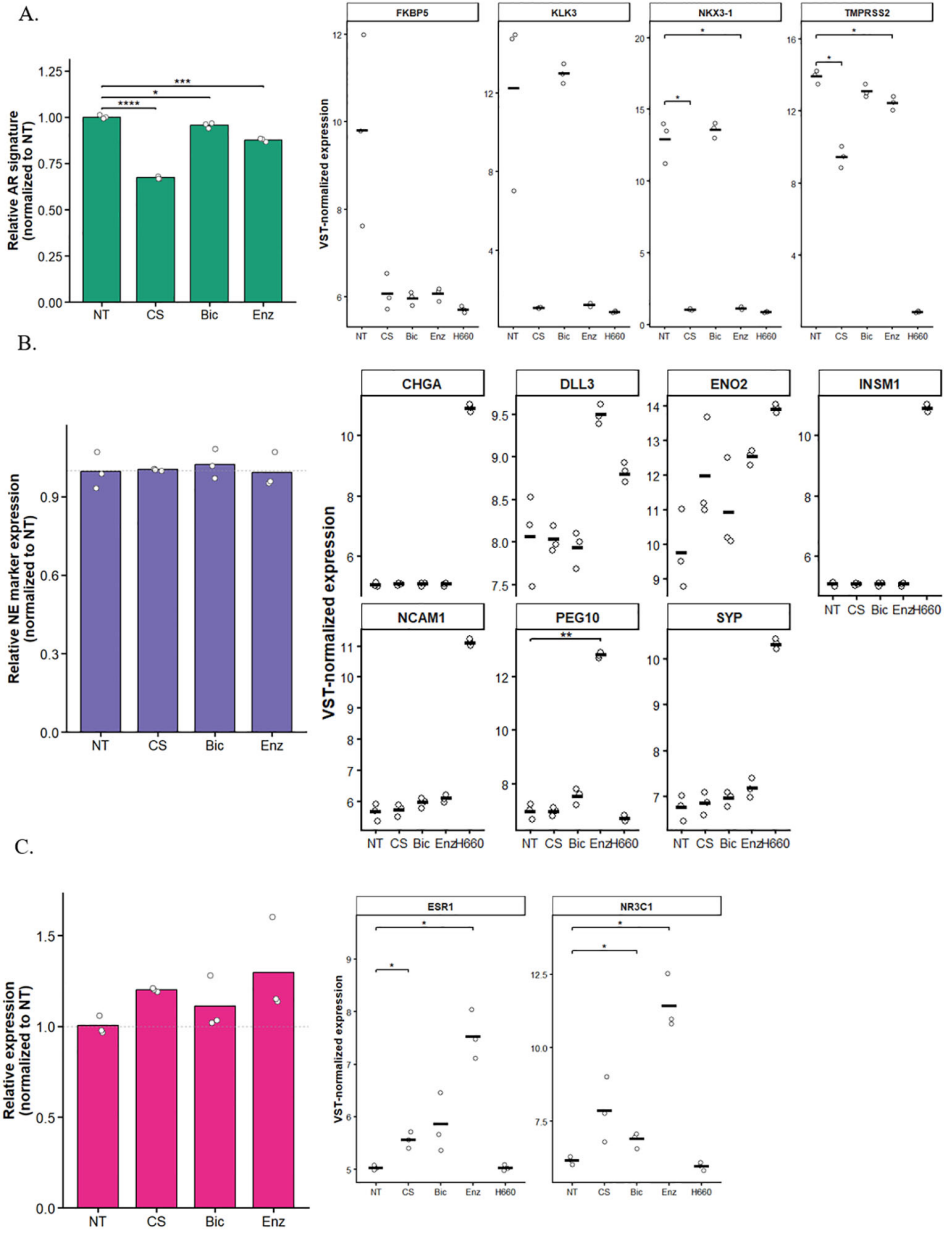
Distinct transcriptional responses to androgen receptor inhibition in LNCaP cells. **(A)** Androgen receptor (AR)–associated gene expression. Left: relative expression of an AR gene signature normalized to no treatment (NT). Statistical comparisons were performed between NT and each treatment condition (CS, Bic, and Enz). Right: VST-normalized expression levels of representative AR target genes (FKBP5, KLK3, NKX3-1, and TMPRSS2) under no treatment (NT), charcoal-stripped serum (CS), bicalutamide (Bic), and enzalutamide (Enz) conditions. The neuroendocrine prostate cancer cell line H660 is shown as a reference. **(B)** Neuroendocrine (NE)–associated gene expression. Left: relative expression of a composite NE marker signature normalized to NT. Statistical comparisons were performed between NT and each treatment condition. Right: VST-normalized expression of individual NE-associated genes (ENO2, NCAM1, DLL3, PEG10, CHGA, SYP, and INSM1) across the indicated conditions. H660 cells are shown as a neuroendocrine reference. **(C)** Expression of genes associated with alternative signaling pathways. Left: relative expression of pathway-associated gene sets normalized to NT. Statistical comparisons were performed between NT and each treatment condition. Right: VST-normalized expression of representative genes (ESR1 and NR3C1) across the indicated conditions. Data points represent individual biological replicates (n = 3). Bars indicate mean values. Statistical comparisons were performed using two-sided unpaired t-tests followed by Benjamini–Hochberg correction for multiple testing. H660 cells were used as a reference NEPC cell line and were not included in statistical testing. For panel A, adjusted P values for NT versus CS, Bic, and Enz were P = 0.094, P = 0.094, and P = 0.098, respectively. For panel B, adjusted P values for NT versus CS, Bic, and Enz were P = 0.955, P = 0.955, and P = 0.955, respectively. For panel C, adjusted P values were ESR1: NT versus CS, P = 0.017 and NT versus Enz, P = 0.010; NR3C1: NT versus Bic, P = 0.022 and NT versus Enz, P = 0.009. *P < 0.05, **P < 0.01, ***P < 0.001, ****P < 0.0001.

AR-associated genes (FKBP5, KLK3, NKX3-1, and TMPRSS2), neuroendocrine-associated genes (ENO2, NCAM1, DLL3, PEG10, CHGA, SYP, and INSM1), and alternative pathway-associated genes (ESR1 and NR3C1) were selected based on established biological relevance and prior literature and were used for visualization and comparative analyses shown in **Figure 2**.

### analyse Gene-wise comparative s and directional shift assessment

To assess gene-wise differences between treatment conditions, pairwise comparisons were performed between NT, Bic, Enz, and H660 samples using DESeq2. Gene-level log_2_ fold changes were calculated for Bic vs NT, Enz vs NT, and H660 vs NT.

Scatter plot analyses were used to compare transcriptional responses between bicalutamide and enzalutamide at the gene level. Genes preferentially induced by enzalutamide were defined based on relative fold-change differences between Enz vs NT and Bic vs NT comparisons.

To characterize genes exhibiting a stepwise transcriptional shift across treatment conditions, genes were ranked based on their relative expression changes from NT to Enz and from Enz to H660. This ranking-based approach enabled identification of genes showing progressive amplification across NT, CS, Bic, Enz, and H660 conditions without presupposing neuroendocrine differentiation.

For gene-wise comparative visualization, no explicit fold-change cutoff was applied unless otherwise stated, and transcriptional responses were interpreted based on relative positional relationships between treatment conditions.

### Functional enrichment analysis

Functional enrichment analysis was performed using Gene Ontology (GO) Biological Process annotations. Genes preferentially induced by enzalutamide were subjected to GO enrichment analysis using the g:Profiler framework with false discovery rate (FDR) correction.

Enriched GO terms were grouped into functional categories related to stimulus response and signaling regulation, developmental and lineage plasticity, and cell migration or morphogenesis. Where applicable, GO intersection genes were extracted and mapped back onto gene-wise scatter plots to evaluate pathway-level trends.

Representative gene sets for lineage plasticity, DNA damage response, metabolic processes, and chromatin or epigenetic regulation were defined based on Gene Ontology annotations and prior literature and were used for visualization in gene-wise comparative analyses.

### Statistical analysis and data visualization

All statistical analyses were conducted in R. Data visualization was performed using ggplot2 and associated packages. PCA was performed using VST-normalized expression values. Sample-to-sample distance heatmaps were generated using Euclidean distance metrics. Boxplots and scatter plots represent biological replicates, and summary statistics are reported as appropriate.

All statistical analyses and data visualizations were performed using R software (version 4.5.1).

## Results

### Enzalutamide treatment induces a global, stepwise transcriptomic shift along an AR suppression–associated continuum in LNCaP cells

To determine whether progressive androgen receptor (AR) inhibition induces global transcriptomic reprogramming, we performed RNA sequencing of androgen-dependent LNCaP cells cultured under stepwise AR-suppressive conditions: untreated (NT), charcoal-stripped serum (CS), bicalutamide (Bic), or enzalutamide (Enz), with the neuroendocrine prostate cancer (NEPC) cell line H660 included as a reference (**Figure 1A**).

Sample-to-sample distance analysis confirmed replicate consistency (**Figure 1B**). H660 samples formed a distinct cluster, whereas CS-, Bic-, and Enz-treated LNCaP samples aligned along a continuum distinct from untreated cells, consistent with progressive transcriptomic remodeling under increasing AR suppression.

Principal component analysis (PCA) confirmed this structure, with the first principal component (PC1) capturing the majority of transcriptomic variance and corresponding to the degree of AR suppression (**Figure 1C**). Bic- and Enz-treated samples distributed along PC1 relative to NT samples, with Enz-treated cells exhibiting increased dispersion, indicating heterogeneous adaptive responses. Although one replicate in several treatment groups showed modest separation in PCA space, sample-to-sample distance analysis demonstrated overall clustering according to treatment condition, suggesting that the observed variation reflects biological heterogeneity rather than a technical artifact.

To relate this shift to lineage-associated programs, we examined AR-regulated and neuroendocrine-associated genes. Canonical AR target genes (KLK3, TMPRSS2, NKX3-1, and FKBP5) were significantly suppressed by androgen deprivation and pharmacologic AR inhibition, whereas their expression was largely absent in H660 cells (**Figure 2A**). In contrast, several neuroendocrine-associated genes, including NCAM1, ENO2, PEG10, and DLL3, showed increased expression under AR-targeted treatment, with higher levels in H660 cells (**Figure 2B**). However, the composite neuroendocrine signature score was not significantly altered, and canonical neuroendocrine differentiation markers, including CHGA, INSM1, and SOX2, were not robustly induced in enzalutamide-treated LNCaP cells.

Because these changes were not fully explained by AR suppression alone, we examined alternative nuclear receptor signaling. ESR1 and NR3C1 (glucocorticoid receptor) showed condition-dependent increases in expression under AR-suppressive conditions, although pathway-level changes remained modest. Their expression patterns were distinct from those observed in H660 cells (**Figure 2C**).

### Enzalutamide disrupts luminal differentiation and induces a transcriptionally plastic intermediate state in LNCaP cells

**Figure 3:**
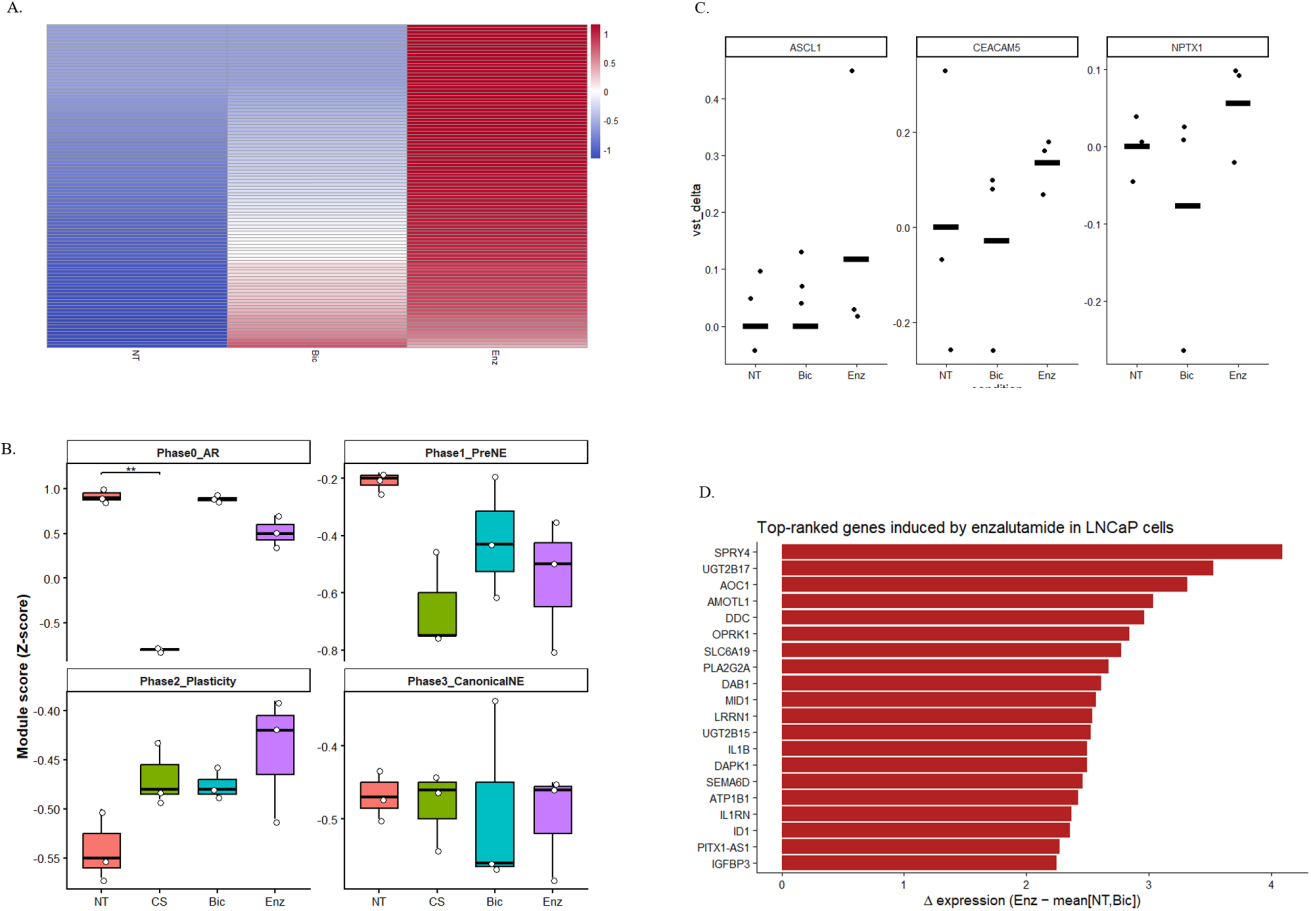
Enzalutamide disrupts luminal differentiation and promotes a plastic transcriptional state in LNCaP cells. **(A)** Heatmap showing genes induced by enzalutamide treatment in LNCaP cells. Gene expression values were averaged across biological replicates for untreated (NT), bicalutamide-treated (Bic), and enzalutamide-treated (Enz) conditions and row-scaled (Z-score) within LNCaP-derived samples. Genes are ordered according to their relative expression in enzalutamide-treated cells. The top 100 ranked genes used for this heatmap analysis are provided in **Supplementary Table 1**. **(B)** Phase-specific module scores (Phase 0–3) across LNCaP-derived conditions. Module scores were calculated using predefined lineage-associated gene sets and are shown as Z-scores. Statistical comparisons were performed between NT and each treatment condition using two-sided unpaired t-tests followed by Benjamini–Hochberg correction. Only significant comparisons are shown. **P < 0.01. The complete module gene sets are provided in **Supplementary Table 2**. **(C)** Expression of representative Phase 2–associated genes (ASCL1, CEACAM5, and NPTX1). Expression values are shown as ΔVST relative to the mean expression in NT samples. Statistical comparisons were performed between NT and each treatment condition using two-sided unpaired t-tests followed by Benjamini–Hochberg correction. Adjusted P values for NT versus Enz were 0.654 for ASCL1, 0.894 for CEACAM5, and 0.654 for NPTX1; no comparison remained statistically significant after multiple-testing correction. **(D)** Genes induced by enzalutamide in LNCaP cells. Genes were ranked based on the difference between enzalutamide-treated samples and the mean of untreated and bicalutamide-treated samples (ΔEnz − mean[NT,Bic]). Bar length indicates the magnitude of differential expression. The complete ranked gene list is provided in **Supplementary Table 3**.

Because this analysis was designed to compare transcriptional differences among NT, Bic, and Enz conditions, the CS group was not included in the **Figures 3A** heatmap.

Global transcriptomic analyses demonstrated that enzalutamide-treated LNCaP cells undergo a directional shift along the transcriptomic continuum defined by progressive AR suppression (Result 1). To define the gene-level programs underlying this shift, we focused on transcriptional changes within LNCaP-derived conditions without presupposing overt neuroendocrine differentiation.

Genes preferentially induced by enzalutamide relative to untreated (NT) and bicalutamide-treated (Bic) LNCaP cells were identified. Heatmap analysis revealed a coordinated transcriptional shift specific to enzalutamide treatment, with enzalutamide-treated cells exhibiting a distinct expression pattern compared with NT and Bic conditions (**Figure 3A**).

To assess differentiation-associated programs, we performed phase-specific module score analysis using predefined differentiation programs (Phase 0–3). Enzalutamide selectively enhanced the early neuroendocrine-like plasticity program (Phase 2), whereas the canonical neuroendocrine differentiation program (Phase 3) was not activated (**Figure 3B**), indicating induction of a non-terminal plastic transcriptional state.

At the individual gene level, representative Phase 2–associated genes, including ASCL1, CEACAM5, and NPTX1, showed modest but reproducible upregulation following enzalutamide treatment compared with NT and Bic conditions (**Figure 3C**).

To quantify enzalutamide-responsive genes, genes were ranked based on differential expression between enzalutamide-treated cells and the mean of NT and Bic conditions (ΔEnz − mean[NT,Bic]). This identified a subset of genes selectively induced by enzalutamide (**Figure 3D**).

### Enzalutamide selectively enhances stimulus-responsive and developmental transcriptional programs in LNCaP cells

**Figure 4:**
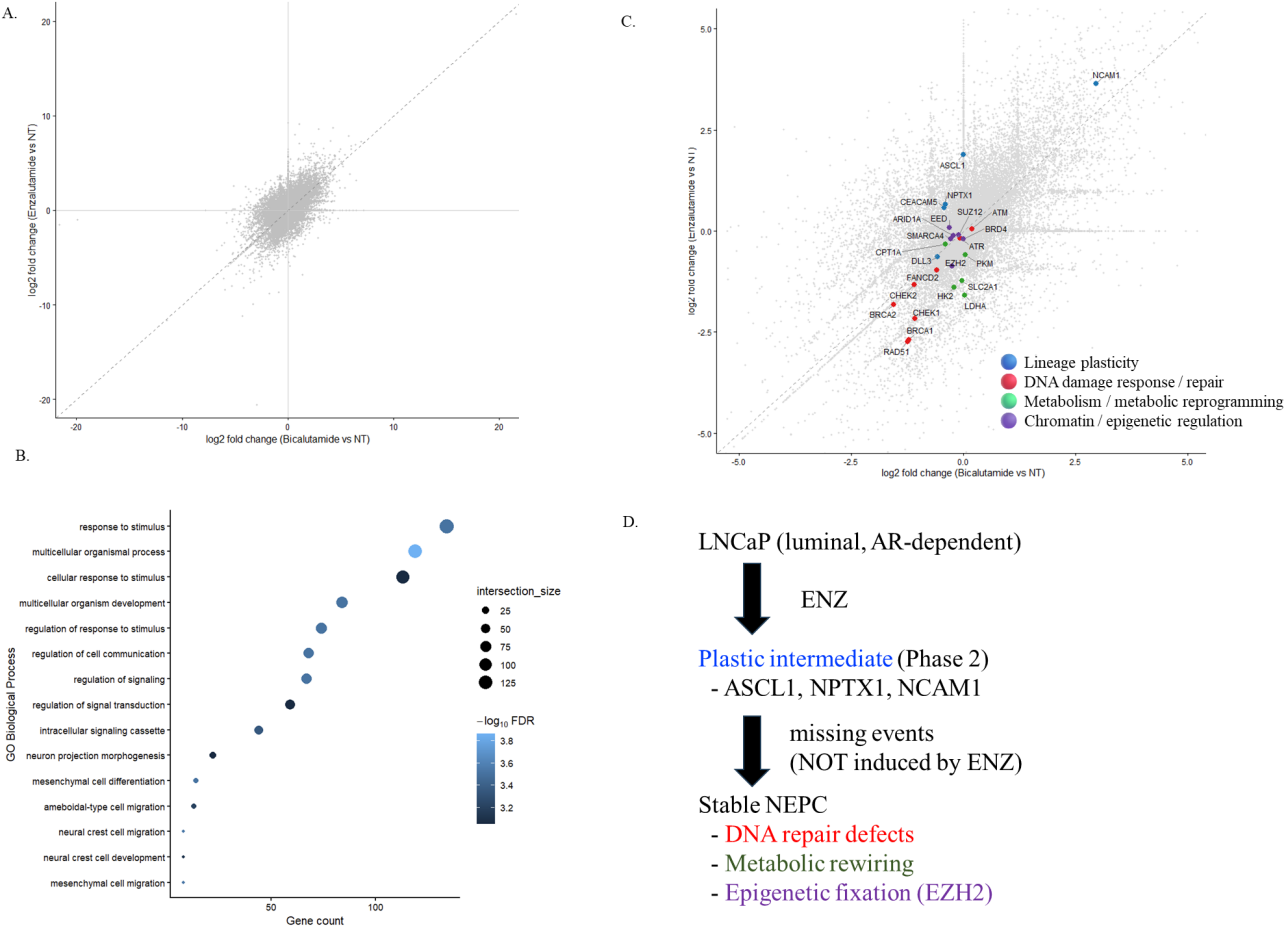
Gene-wise transcriptional features associated with enzalutamide treatment. **(A)** Gene-wise comparison of transcriptional responses to bicalutamide and enzalutamide. Scatter plot shows log2 fold changes relative to untreated LNCaP cells. Dashed line indicates y = x, and solid grey lines indicate no change (log2 fold change = 0) under each treatment. **(B)** Functional enrichment analysis of genes relatively preferentially induced by enzalutamide compared with bicalutamide. Enriched Gene Ontology biological processes highlight stimulus responsiveness and lineage plasticity–related programs. **(C)** Overlay of representative gene sets on the gene-wise comparison. Genes associated with Phase 2 lineage plasticity, DNA damage response, metabolic reprogramming, and chromatin/epigenetic regulation are highlighted. Individual gene symbols are shown for all genes included in each category. **(D)** Conceptual summary of transcriptional states induced by enzalutamide. Enzalutamide promotes a plastic intermediate state, while transcriptional programs required for stable neuroendocrine commitment, including DNA repair defects, metabolic rewiring, and epigenetic fixation, are not activated.

To delineate transcriptional programs underlying the enzalutamide-associated shift, we compared gene-wise responses between enzalutamide- and bicalutamide-treated LNCaP cells. Gene-wise scatter plot analysis showed broadly correlated responses to both androgen receptor antagonists, with a limited subset of genes exhibiting preferential induction by enzalutamide (**Figure 4A**).

Gene Ontology enrichment analysis of enzalutamide-preferential genes revealed enrichment of stimulus-responsive, signaling, and developmental or lineage-associated processes (**Figure 4B**).

In contrast, genes associated with canonical DNA damage response, metabolic reprogramming, and chromatin or epigenetic regulation did not preferentially localize to the enzalutamide-dominant region and showed largely unchanged or heterogeneous responses (**Figure 4C**).

Although migration- and morphogenesis-related Gene Ontology terms were enriched, classical migration- or epithelial–mesenchymal transition–associated marker genes were not robustly induced, with only modest and coordinated expression changes.

By contrast, a small set of developmental and lineage plasticity–associated genes, including ASCL1, NKX2-1, NCAM1, and NPTX1, preferentially localized to the enzalutamide-dominant region and overlapped with genes contributing to the H660-oriented transcriptional shift observed previously (**Figures 3**, **4C**).

Together, these results indicate selective enhancement of stimulus-responsive and developmental plasticity–associated transcriptional programs by enzalutamide in LNCaP cells (**Figure 4D**).

### Context-dependent transcriptional plasticity induced by partial and potent AR inhibition in C4–2 cells

**Figure 5:**
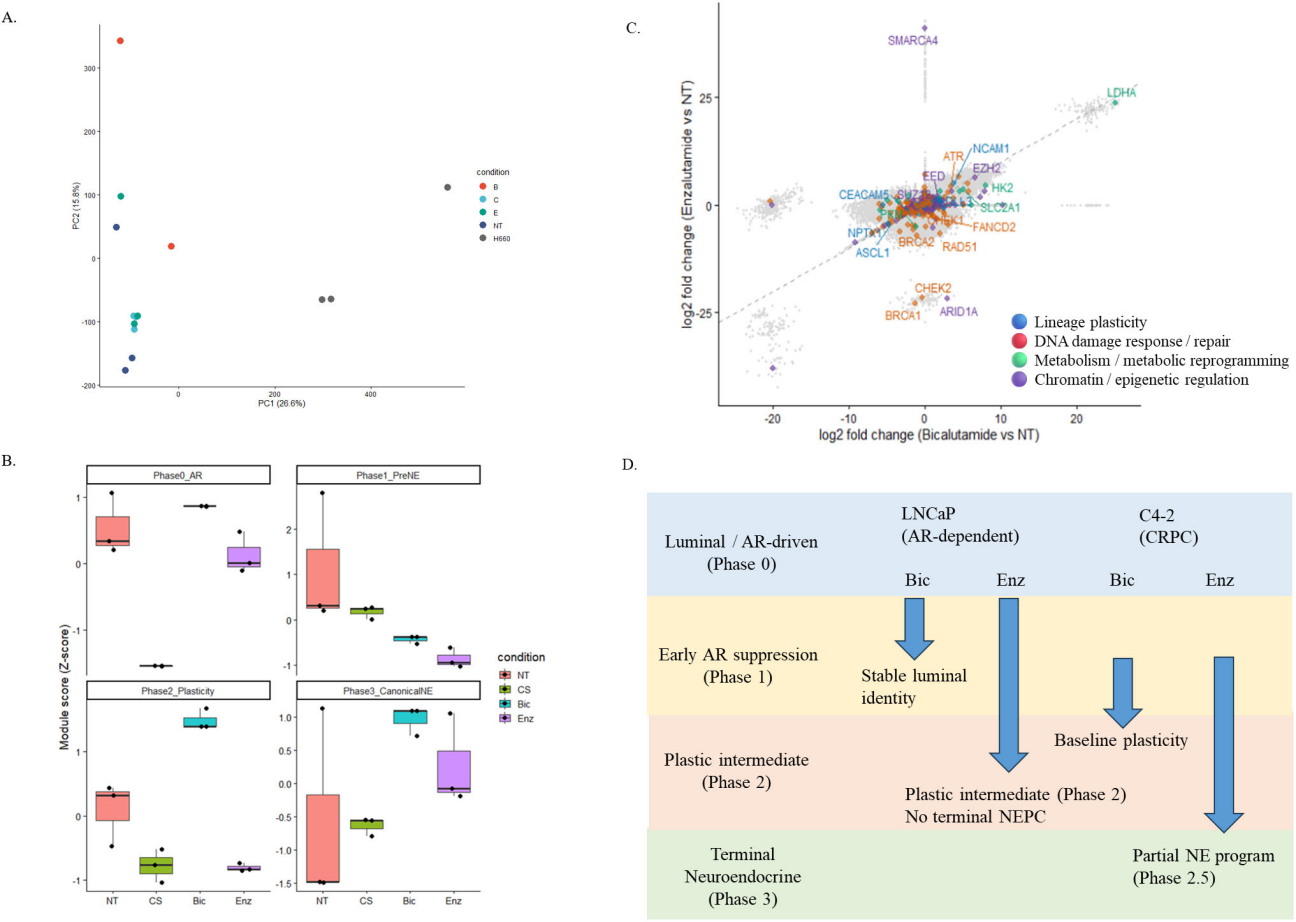
Transcriptional responses to AR inhibition in C4–2 cells. **(A)** Principal component analysis (PCA) of C4–2 cells under different AR-suppressive conditions together with the neuroendocrine prostate cancer cell line H660. **(B)** Module score analysis of transcriptional programs representing AR signaling (Phase 0), pre-neuroendocrine transition state (Phase 1), lineage plasticity/chromatin remodeling program (Phase 2), and canonical neuroendocrine differentiation (Phase 3) in C4–2 cells under untreated control (NT), charcoal-stripped serum (CS), bicalutamide (Bic), and enzalutamide (Enz) conditions. Module scores were calculated using the gene sets listed in **Supplementary Table 2** and are shown as Z-scores. **(C)** Scatter plot showing gene-level log_2_ fold changes in C4–2 cells treated with bicalutamide (Bic; x-axis) or enzalutamide (Enz; y-axis) relative to untreated controls (NT). Selected genes related to neuroendocrine differentiation, DNA damage response, epigenetic regulation, and metabolism are highlighted. **(D)** Proposed model summarizing distinct transcriptional responses to long-term AR suppression in AR-dependent LNCaP and castration-resistant C4–2 cells. Whereas LNCaP cells transition from a luminal AR-driven state toward a plastic intermediate state without terminal neuroendocrine differentiation, C4–2 cells appear to maintain a pre-existing plastic transcriptional state and exhibit only partial engagement of neuroendocrine-associated programs following additional AR suppression.

To determine how different modes of androgen receptor (AR) inhibition shape transcriptional states in castration-resistant prostate cancer, we analyzed RNA-seq profiles of C4–2 cells treated with charcoal-stripped serum, bicalutamide, or enzalutamide. Principal component analysis showed that C4–2 cells remained transcriptionally distinct from the neuroendocrine prostate cancer cell line H660 under all conditions, while both bicalutamide- and enzalutamide-treated samples exhibited an incomplete directional shift toward a neuroendocrine-like transcriptional space (**Figure 5A**). To assess pathway-level changes, we quantified module scores representing AR signaling (Phase 0), pre-neuroendocrine state (Phase 1), lineage plasticity (Phase 2), and canonical neuroendocrine differentiation (Phase 3). Consistent with the castration-resistant phenotype, AR-associated transcriptional programs were not uniformly suppressed by AR-targeted therapies (**Figure 5B**), and pre-neuroendocrine programs were not progressively induced. Lineage plasticity–associated programs were detectable across all conditions, consistent with a pre-existing plastic transcriptional state in C4–2 cells. Canonical neuroendocrine programs showed modest changes following AR suppression, with evidence of partial engagement of neuroendocrine-associated transcriptional programs. Unlike LNCaP cells, however, these changes occurred in the setting of pre-existing plasticity-associated transcriptional features and were not accompanied by progressive activation of pre-neuroendocrine programs.

Gene-level comparison of transcriptional responses to bicalutamide and enzalutamide revealed distinct effects of partial versus potent AR inhibition (**Figure 5C**). While a subset of genes showed concordant regulation, key regulators of epigenetic remodeling, DNA damage response, metabolism, and neuroendocrine differentiation exhibited divergent expression patterns between the two treatments.

Collectively, these results indicate that C4–2 cells, as a castration-resistant prostate cancer model, already occupy a permissive transcriptional landscape characterized by partial plasticity-associated features. In this context, AR suppression appeared to modulate pre-existing transcriptional states rather than induce a progressive transition toward NEPC. Although both bicalutamide and enzalutamide influenced neuroendocrine-associated transcriptional programs, neither treatment resulted in stable canonical neuroendocrine differentiation.

## Discussion

In this study, we conceptualized treatment-emergent lineage plasticity as a stepwise framework spanning transcriptomic states from Phase 0 to Phase 3. Phase 0–1 represent AR-dependent adenocarcinoma states with intact AR signaling and preserved luminal identity despite early AR suppression (3, 11). Phase 2 represents a transitional state marked by destabilization of luminal differentiation and selective activation of lineage plasticity–associated transcriptional programs (12, 13). For example, ONECUT2 has been reported as a plasticity-associated transcription factor shared across adenocarcinoma and neuroendocrine prostate cancer states; however, its activation alone is insufficient to drive terminal neuroendocrine differentiation (12).

Phase 3 corresponds to bona fide NEPC, defined by stable engagement of neuroendocrine differentiation programs, frequent loss of key tumor suppressors such as RB1 and TP53, and accompanying molecular features including DNA repair defects, metabolic rewiring, and epigenetic alterations (4, 7, 14–17).

In ARPC, modeled by LNCaP cells, potent AR pathway inhibition was associated with features consistent with Phase 2, including upregulation of lineage plasticity–associated transcriptional programs. Despite this shift, LNCaP cells did not activate Phase 3–defining neuroendocrine programs or acquire molecular features of bona fide NEPC, remaining within a plastic but non-neuroendocrine state.

Importantly, enzalutamide-treated LNCaP cells consistently exhibited transcriptional features corresponding to Phase 2, including activation of developmental and lineage plasticity–associated programs, while lacking stable Phase 3 neuroendocrine differentiation. These findings support the concept that potent AR pathway inhibition promotes an early adaptive plastic state that precedes, but does not itself constitute, bona fide NEPC.

In contrast, CRPC, represented by C4–2 cells, displayed a distinct pattern of transcriptional response. Lineage plasticity–associated transcriptional features were detectable across treatment conditions, suggesting that C4–2 cells already reside in a permissive plastic transcriptional state. Additional AR suppression produced only modest changes in these programs, indicating modulation of a pre-existing plastic state rather than *de novo* induction of lineage plasticity.

Accordingly, both partial and potent AR inhibition permitted partial activation of Phase 3–associated neuroendocrine transcriptional programs in CRPC cells. This activation was heterogeneous and incomplete, indicating that CRPC cells display transcriptional features compatible with neuroendocrine differentiation but lack molecular events required for irreversible commitment. Thus, AR inhibition in CRPC appears to function less as a strictly linear driver of lineage transition and more as a contextual modifier shaping accessible transcriptional states.

Taken together, these findings indicate that ARPC and CRPC differ in their responses to AR pathway perturbation. While ARPC cells demonstrated transcriptional changes consistent with Phase 2 under potent AR inhibition, CRPC cells exhibited a permissive intermediate context in which both partial and potent AR inhibition elicited incomplete neuroendocrine transcriptional programs without commitment to terminal NEPC.

These observations highlight that potent AR pathway inhibition preferentially promotes lineage plasticity–associated transcriptional programs in AR-dependent prostate cancer cells. In LNCaP cells, enzalutamide induced a plastic intermediate state characterized by partial activation of neuroendocrine-associated transcriptional programs compared with bicalutamide. However, this response did not represent terminal neuroendocrine differentiation. In contrast, C4–2 cells, as a CRPC model, appeared to reside within a pre-existing permissive plastic transcriptional state, and additional AR suppression primarily modified this baseline state rather than inducing a LNCaP-like stepwise transition.

To integrate these observations, we propose a conceptual model in which treatment-induced lineage plasticity is influenced by interactions between tumor context and the qualitative nature of AR inhibition (**Figure 5D**). In AR-dependent prostate cancer, potent AR blockade destabilizes luminal identity and is associated with features consistent with a plastic intermediate transcriptional state, whereas CRPC cells appear to reside within a pre-existing plastic transcriptional landscape with accessible lineage-associated programs. Within this context, AR suppression primarily modifies existing transcriptional states and permits partial engagement of neuroendocrine-associated programs without evidence of irreversible commitment to terminal NEPC.

Treatment-emergent lineage plasticity is unlikely to be determined solely by tumor cell–intrinsic transcriptional changes. Therapy-induced remodeling of the tumor microenvironment, including stromal WNT16B–mediated paracrine signaling, may facilitate accumulation of additional genetic or epigenetic alterations required for stable neuroendocrine commitment (10, 18–21).

Consistent with this notion, spatial transcriptomic analyses of human NEPC specimens have shown that neuroendocrine tumor regions are surrounded by distinct microenvironments characterized by fibroblast activation and immune exclusion, supporting a critical role for tumor–microenvironment interactions in NEPC development (22). As the present study is based on *in vitro* cell line models, it cannot fully capture intratumoral heterogeneity or microenvironmental influences. In addition, because our analyses were performed using bulk RNA sequencing at the experimental endpoint, we were unable to determine whether the observed transcriptional changes reflected *de novo* transcriptional reprogramming within individual cells or selective expansion of pre-existing cellular subpopulations. Recent advances in single-cell and spatial transcriptomics have demonstrated the ability to reconstruct lineage trajectories and resolve treatment-associated cellular states at high resolution, providing a framework to distinguish transcriptional adaptation from cellular selection (23, 24). Further studies integrating patient-derived xenograft or organoid models with single-cell transcriptomic analyses will be necessary to clarify how adaptive transcriptional states relate to clinically established NEPC (25, 26). Whether the observed transcriptional changes are functionally required for adaptation, lineage plasticity, or neuroendocrine-associated state transitions also remains unclear. Functional validation using genetic perturbation approaches, lineage-tracing experiments, and patient-derived model systems will be necessary to determine the biological significance of these candidate gene programs.

It should also be recognized that the apparent increase in NEPC in the era of potent AR pathway inhibition may reflect not only true biological transformation but also enhanced clinical recognition enabled by repeat biopsies, molecular profiling, and refined pathological criteria. Nevertheless, the emergence of AR-independent or neuroendocrine-associated phenotypes during therapy remains a clinically meaningful problem warranting continued mechanistic investigation. These issues should be considered in the broader context of recent advances in prostate cancer biology, evolving pathological classifications of neuroendocrine differentiation and aggressive histologic subtypes, and molecular mechanisms of treatment resistance and DNA damage repair (27–30).

In summary, the present study positions transcriptional reprogramming induced by AR pathway inhibition, including enzalutamide, as an early adaptive response within treatment-emergent lineage plasticity rather than evidence of terminal neuroendocrine differentiation. Enzalutamide does not directly induce stable NEPC differentiation in ARPC models but, compared with bicalutamide, more strongly promotes transcriptional plasticity characterized by partial activation of neuroendocrine- and plasticity-associated gene programs. Although next-generation AR pathway inhibitors remain indispensable in advanced prostate cancer, our findings underscore the need to better understand how sustained therapeutic pressure shapes prostate cancer evolution.

## Data Availability

The datasets presented in this study can be found in online repositories. The names of the repository/repositories and accession number(s) can be found below: https://www.ncbi.nlm.nih.gov/geo/, GSE315550.

## Supplementary material

Supplementary Table 1 Top 100 genes used for the **Figure 3A** heatmap analysis. Genes were ranked according to the directional score calculated from gene expression changes across NT, Enzalutamide-treated LNCaP, and H660 cells. The top 100 ranked genes displayed in **Figure 3A** are listed together with their expression values.

Supplementary Table 2 Gene sets used for phase-specific module score calculation. Gene signatures used to calculate the Phase0_AR, Phase1_PreNE, Phase2_Plasticity, and Phase3_CanonicalNE module scores shown in **Figure 3B**. Module scores were calculated using the average normalized expression of genes included in each signature.

Supplementary Table 3 Complete ranked gene list used for the **Figures 3D analysis**. Genes were ranked according to differential expression between Enzalutamide-treated cells and the mean of untreated and bicalutamide-treated cells (ΔEnz − mean[NT,Bic]). The complete ranked gene list is provided.
